# Ethnic disparities in insulin and glucose-dependent insulinotropic peptide (GIP) responses to intraduodenal glucose in health

**DOI:** 10.1007/s00592-014-0684-x

**Published:** 2014-11-16

**Authors:** Chinmay S. Marathe, Michelle Bound, Kylie Lange, Karen L. Jones, Christopher K. Rayner, Michael Horowitz

**Affiliations:** 1 Discipline of Medicine, Royal Adelaide Hospital, University of Adelaide, Adelaide, 5000 Australia; 2 Centre of Research Excellence (CRE) in Translating Nutritional Science to Good Health, University of Adelaide, Adelaide, Australia

East Asians appear to secrete less insulin than Caucasians following oral glucose suggesting that impaired insulin secretion is fundamental to the pathogenesis of type 2 diabetes [1]. Information about the secretion of the incretin hormones, GIP and GLP-1, dependent on duodenal glucose load [2], in East Asians is limited [3]. We have evaluated glycemic, insulinemic and incretin hormone responses to intraduodenal glucose in healthy Han Chinese.

We studied eleven Han Chinese (HC) and eight Caucasian (C) healthy men; the latter included in a previous study [2]. Each subject attended following an overnight fast. A catheter, incorporating an infusion channel opening 12 cm beyond the pylorus, was inserted intranasally [2]. An IV cannula was placed in an antecubital vein. Intraduodenal (ID) glucose (25 g/100 mL) was infused at 4 kcal/min from *t* = 0 to 120 min. Blood was collected at *t* = 0, 15, 30, 45, 60, 90, 105 and 120 min for measurements of blood glucose, plasma insulin, GIP and GLP-1. Insulin secretion was estimated as the change in insulin divided by the change in glucose at 30 min (∆*I*_0–30_/∆*G*_0–30_). Insulin sensitivity was estimated as 1/fasting insulin. The disposition index (DI_O_) was calculated as ∆*I*_0–30_/∆*G*_0–30_ *X* 1/fasting insulin. Unpaired Student’s *t* test was used in analysis.

Han Chinese younger than Caucasians (24.8 ± 1.3 HC vs. 45.3 ± 3.8 C years, *P* < 0.01); there was no difference in BMI (25.1 ± 1.7 HC vs. 28.3 ± 0.7 C kg/m^2^). There were no differences in fasting glucose (5.4 ± 0.1 HC vs. 5.7 ± 0.2 C, mmol/L, *P* = 0.10) or glycemic response to ID glucose. Fasting (4.9 ± 0.8 HC vs. 19.2 ± 3.9 C, mU/L, *P* < 0.01) and AUC_0–120_ (13,234 ± 2,134 HC vs. 43,133 ± 12,197 C, mU/L min, *P* = 0.01) insulin and insulin secretion (15.5 ± 5.2 HC vs. 63.2 ± 22 C, *P* = 0.02) were lower in Han Chinese. The DI_O_ was not different (2.9 ± 0.4 HC vs. 3.5 ± 1.3 C, *P* = 0.63). Fasting (16.2 ± 1.3 HC vs. 22 ± 2.9 C, pmol/L, *P* = 0.06) and AUC_0–120_ (5,836 ± 337 HC vs. 7,975 ± 739 C, pmol/L min, *P* = 0.01) GIP were lower in Han Chinese. There was no difference in fasting (25 ± 3.3 HC vs. 19.8 ± 2.4 C, pmol/L, *P* = 0.24) or glucose-stimulated GLP-1 (Fig. 1).

**Fig. 1:**
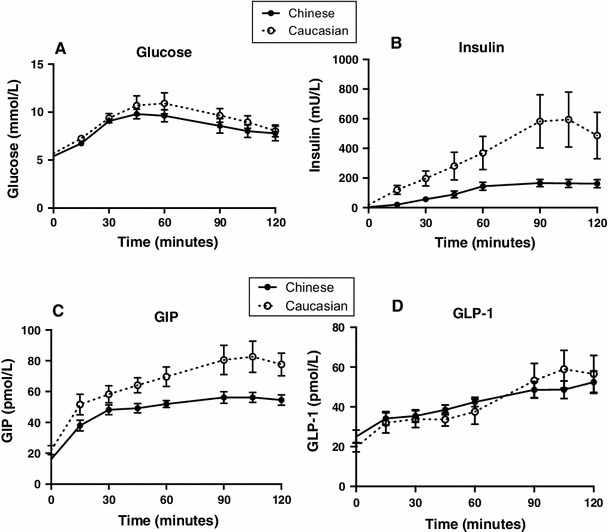
Blood glucose (**a**), plasma insulin (**b**), GIP (**c**) and GLP-1 (**d**) concentrations at baseline and in response to a 120-min intraduodenal glucose infusion at 4 kcal/min in Han Chinese (*Filled circles with bold line*) and Caucasian (*Empty circles with dotted line*) subjects. Data are mean ± SEM

Our study indicates that, in response to intraduodenal glucose infusion, insulin secretion is less and insulin sensitivity is greater in Han Chinese than in Caucasians, associated with reduced GIP, but comparable GLP-1, secretion and DI_O_—the latter reflecting increased insulin sensitivity in Han Chinese. Few studies have evaluated GIP and GLP-1 responses within East Asian populations. In the only direct comparison [3], healthy Japanese were reported to have higher GIP and lower GLP-1 than Caucasians, but methodological limitations preclude meaningful interpretation. The reduced GIP response we observed could contribute to the diminished insulin response. In type 2 diabetes, the insulinotropic capacity of GIP is markedly reduced, and the reduction in GIP is likely to be of primary relevance to ‘health.’

Limitations of our study are that the cohort was of small size and exclusively male, that responses to intraduodenal, rather than oral, glucose were evaluated and that there was a difference in age between the groups, although GIP (and GLP-1) response is apparently unaffected by age [4]. Mean BMI was higher in the Caucasians, albeit non-significantly, which may represent a confounder, although it appears that body weight does not affect the GIP response to nutrients [5].

## Abbreviations

ID4: Intraduodenal glucose infusion at 4 kcal/min
GIP: Glucose-dependent insulinotropic polypeptide
GLP-1: Glucagon-like peptide-1
DI: Disposition index
HC: Han Chinese
C: Caucasian
